# Policy Considerations for Using Forests to Mitigate Carbon Dioxide Emissions

**DOI:** 10.1100/tsw.2001.60

**Published:** 2001-06-16

**Authors:** Sandra Brown, R. Neil Sampson, Bernhard Schlamadinger, John Kinsman

**Affiliations:** Winrock International, c/o 831 NW Sundance Circle, Corvallis, OR 97330, USA; The Sampson Group, Inc., 5209 York Road, Alexandria, VA 22310, USA; Joanneum Research, Elisabethstrasse 5, A-8010 Graz, Austria; Edison Electric Institute, 701 Pennsylvania Avenue, N.W., Washington, D.C. 20004, USA

**Keywords:** carbon sequestration, afforestation, reforestation, forests, CO_2_ fertilization, greenhouse gases, climate change, Kyoto Protocol

## Abstract

A recent article in Nature, “Soil Fertility Limits Carbon Sequestration by Forest Ecosystems in a CO2-Enriched Atmosphere” by Oren and colleagues[1], has been widely reported on, and often misinterpreted, by the press. The article dampens enthusiasm for accelerated forest growth due to CO2 fertilization and puts in question the fringe theory that the world’s forests can provide an automatic mitigation feedback. We agree that these results increase our understanding of the global carbon cycle. At the same time, their relevance in the context of the international climate change negotiations is much more complicated than portrayed by newspapers such as the New York Times (“Role of Trees in Curbing Greenhouse Gases is Challenged”, May 24, 2001) and the Christian Science Monitor (“Trees No Savior for Global Warming”, May 25, 2001).

To put the findings by Oren et al. in the policy context, a smaller role of $CO_2$ fertilization than previously thought does not mean that trees and forests would be less significant in meeting the Kyoto Protocol. The Protocol is quite explicit in that carbon credits can only accrue due to direct human-induced changes in the management of forests, agricultural, and grazing lands; and that any carbon credits that do accrue must be measurable, transparent, and verifiable. In fact, the IPCC[2] goes to great lengths in discussing how forestry activities could be measured to factor out $CO_2$ fertilization. If indeed such an effect does not exist or is of minor importance, as indicated in the article by Oren et al.[1], then the need to measure control areas to factor out $CO_2$ fertilization will be eliminated.

Additionally, the Oren et al. findings do not undermine the fact that planting new trees where originally there were none (afforestation and reforestation) can remove a substantial amount of $CO_2$ from the atmosphere. Forest protection to keep $CO_2$ out of the atmosphere is equally important since forest destruction and degradation create one quarter of human-induced $CO_2$

emissions to the atmosphere. In addition, better management of existing forest, agricultural, and grazing lands can increase carbon stocks on the land, which will in turn reduce carbon in the atmosphere. It is also important to note that these activities can provide many other environmental benefits, including restoration of degraded lands, preservation/enhancement of wildlife habitat, and improvements in water quality.

It is widely recognized that terrestrial carbon sequestration is not the long-term solution to greenhouse gases and climate change, as has been elaborated on at length in the recent IPCC special report[2]. Also, the IPCC Second Assessment Report has pointed out that all sequestration measures (including avoidance of deforestation) can only offset 12-15% of fossil-fuel emissions in the next 50 or so years[3].

Nevertheless, expanding, protecting, and managing forests remain effective strategies that can complement emission reduction measures in the energy sector.
